# A Case of Localized Prostate Cancer Associated with Polymyalgia Rheumatica with Marked Symptomatic Improvement after Robot-Assisted Radical Prostatectomy

**DOI:** 10.1155/2021/8026883

**Published:** 2021-10-15

**Authors:** Makoto Kawase, Keita Nakane, Sanae Namiki, Yasumichi Takeuchi, Shota Ueda, Kota Kawase, Chie Nakai, Shinichi Takeuchi, Daiki Kato, Manabu Takai, Koji Iinuma, Masayuki Fuwa, Chiemi Saigo, Tatsuhiko Miyazaki, Hiroyuki Morita, Takuya Koie

**Affiliations:** ^1^Department of Urology, Gifu University Graduate School of Medicine, Japan; ^2^Department of General Internal Medicine, Gifu University Graduate School of Medicine, Japan; ^3^Department of Pathology, Gifu University Graduate School of Medicine, Japan

## Abstract

A 73-year-old man visited our hospital with chief complaints of fever of unknown origin and bilateral shoulder and hip joint pain. He was initially diagnosed with polymyalgia rheumatica (PMR). Although the patient was treated with prednisolone 15 mg/day, his PMR-related symptoms did not improve. Further examination was performed as the patient was suspected of having paraneoplastic syndrome. Assessment results showed prostate cancer without metastases. After undergoing robot-assisted radical prostatectomy, the patient's PMR-related symptoms dramatically improved. Hence, the prednisolone dose was decreased to 4 mg/day. PCa may have triggered the development of PMR through the activation of immune-mediated systemic inflammatory responses.

## 1. Introduction

Paraneoplastic syndrome (PNS) is an extremely rare disorder that occurs in <1% of all cancer patients [1]. A paraneoplastic phenomenon usually develops due to the following reasons: (a) biologically active substances aberrantly produced by the underlying neoplasm; (b) modulation of the immune system via autoimmunity, immune complex production, and immunosuppression; and (c) unknown causes [2]. Polymyalgia rheumatica (PMR) is a relatively common inflammatory rheumatological disorder that is frequently associated with a certain type of cancer such as PNS [3]. Recently, three studies have reported the association between PMR and cancer [4–6]. However, it remains unclear whether a true association exists between PMR and malignant diseases, including prostate cancer (PCa), or whether it is a coincidental existence of the two conditions [3]. Thus far, only four prostate cancer patients with PMR who had multiple metastases received androgen deprivation therapy, and their PNS-related symptoms eventually improved without steroid treatment [7, 8]. In this case report, a PCa patient with PMR underwent surgical treatment and showed improvements in PMR-related symptoms.

## 2. Case Presentation

**Figure 1:**
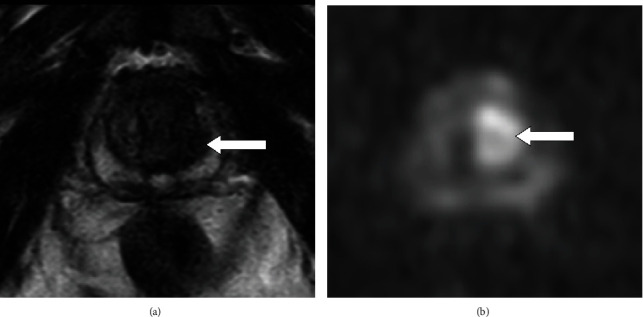
(a) Magnetic resonance imaging showed a low-intensity area in the T2-weighted image (white arrow). (b) A high-intensity area in the diffusion-weighted image at the left transition zone (white arrow).

**Figure 2:**
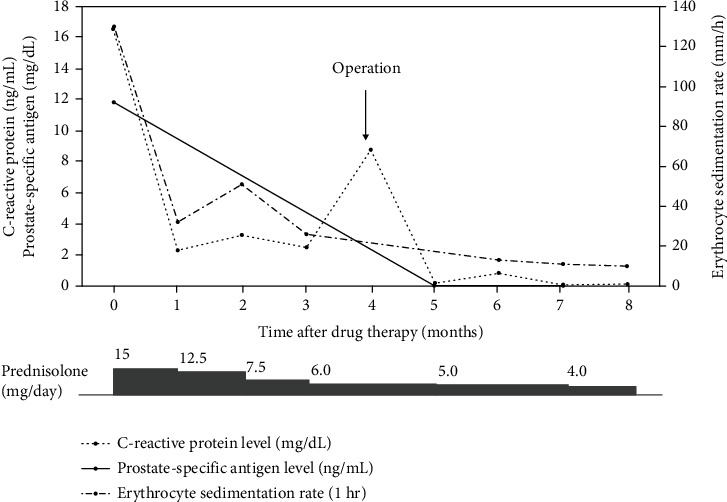
Clinical course and serum levels of an erythrocyte sedimentation rate, a C-reactive protein, and a prostate-specific antigen.

A 73-year-old Japanese man was referred to our hospital due to complaints of fever of unknown origin and bilateral shoulder and hip joint pain. His symptoms had gradually progressed, and he experienced loss of walking ability, general malaise, and lethargy. He was diagnosed with PMR according to the 2012 Provisional Classification Criteria for Polymyalgia Rheumatica [9]. He was treated with 15 mg/day prednisolone. Three months later, the patient's PMR-related symptoms did not improve. Thus, further examinations were performed as the patient was suspected of having PNS. He had a hemoglobin level of 8.0 g/dL (normal range: 13.7–16.8 g/dL), a leukocyte count of 16,140/*μ*L (normal range: 3,300–8,600/*μ*L), an erythrocyte sedimentation rate of 130 (1 h), and a C-reactive protein level of 16.54 mg/dL (normal level: <0.14 mg/dL). His renal, liver, and thyroid test results were normal. The serum prostate-specific antigen level increased to 11.852 ng/mL (normal level: <4.0 ng/dL). Magnetic resonance imaging revealed a low-intensity area in the T2-weighted image and a high-intensity area in the diffusion-weighted image at the left transition zone (Figures 1(a) and 1(b)). The patient was diagnosed with adenocarcinoma of the prostate with a Gleason score of 3 + 4 based on the results of the pathological examination of prostate biopsy specimens. Bone scintigraphy and chest and abdominal computed tomography showed absence of distant metastases and lymph node involvement. Hence, the patient was clinically diagnosed with localized PCa according to the staging system defined in the American Joint Committee on Cancer Staging Manual [10]. Robot-assisted radical prostatectomy was performed. Histopathological findings showed presence of adenocarcinoma of the prostate with T2c stage, a Gleason score of 3 + 4, and a negative surgical margin. There were no neuroendocrine carcinoma components in the specimen. Four months after surgery, the patient's PMR-related symptoms dramatically improved, and he was able to walk without pain. The steroid dose was decreased to 4 mg/day (Figure 2).

## 3. Discussion

The association between cancer and PMR has been established in the following patients: first, patients with a rheumatic disorder, which is directly triggered by a tumor or its metastasis; second, patients with an established rheumatic condition who are at increased risk of specific malignancies; and third, patients with PNS [11]. Currently, PNS is recognized to be caused by antibody production [12] and aberrant hormone release or abnormal cytokine release due to the production of vascular endothelial growth factors [13]. PNS-related symptoms have an impact on several body systems, including the hematological, cutaneous, vascular, and neurological systems [14]. Although the exact cause of PNS in PCa remains unknown, most hypotheses suggest that inappropriate release of peptide hormones, biologic amines, or growth factors is the primary cause [15]. PNS caused by antibody production, aberrant hormone release, or abnormal cytokine is more frequently diagnosed in patients with prostate cancer, which contains small-cell carcinoma components than those of adenocarcinoma [15]. Nguyan et al. reported that interleukin-6 (IL-6) is a key mediator in several stages of the PCa pathogenesis, including initiation of prostate tumorigenesis, stimulation of tumor growth, induction of aggressive PCa phenotype, PCa progression to the castration-resistant state, promotion of tumor metastasis, and resistance to chemotherapy [16]. Although IL-6 may have an important role in the progression of PMR to PNS in patients with PCa, limited evidence suggests that PCa is commonly diagnosed with incident PMR [4].

According to a previous large cohort study, which enrolled 35,918 patients with PMR and giant cell arteritis (GCA) from Sweden using the Swedish Hospital Discharge Register, 3,941 patients (11.0%) were diagnosed with cancer [4]. Of these patients, 19.1% developed cancer within the first year after the diagnoses of PMR and GCA [4]. Moreover, PMR and GCA patients were frequently diagnosed with skin cancer and acute myeloid leukemia [4]. In 2013, using the General Practice Research Database, Muller et al. reported that older patients with PMR were more likely to be diagnosed with cancer within 6 months after the diagnosis of PMR [5]. Although these patients were treated with at least two different corticosteroid agents, data regarding patients' response to treatment were not available [5]. In a previous systematic review and meta-analysis including six studies, the pooled risk ratio of malignancy in patients with PMR and GCA compared with control patients was 1.14 (95% confidence interval: 1.05–1.22) [6]. The risk of malignancy appeared to be higher in the first 6–12 months after diagnosis [6]. However, the pooled risk ratio decreased to 8% and was not significant [6].

In this case, PCa may have triggered the development of PMR through the activation of immune-mediated systemic inflammatory responses caused by antibody production, aberrant hormone release, or abnormal cytokine. To our knowledge, this is the first case report to describe a PCa patient with PMR who underwent surgical treatment and showed improvements in PMR-related symptoms.

## Data Availability

The data that support the findings of this case report are available from the corresponding author, K.N., upon reasonable request.
